# Differential Antioxidant Enzyme Gene Expression and Functional Analysis of Pyridaben-Susceptible and -Resistant Strains of *Tetranychus truncatus* (Acari: Tetranychidae) under High Temperature Stress

**DOI:** 10.3390/insects15060381

**Published:** 2024-05-23

**Authors:** Liwen Song, Cailan Yu, Wenliang Li, Lei Liu, Qinzhe Sun, Huan Liu, Senshan Wang

**Affiliations:** Biocontrol Engineering Laboratory of Crop Diseases and Pests of Gansu Province, College of Plant Protection, Gansu Agricultural University, Lanzhou 730070, China

**Keywords:** *Tetranychus truncatus*, antioxidant enzyme phylogeny and structure analyses, gene expression patten, high temperature stress, gene function

## Abstract

**Simple Summary:**

*Tetranychus truncatus* (Acari: Tetranychidae) is the dominant pest on many crops in China. The resistant strain cultivated by pyridaben in our laboratory showed higher adaptability to high temperature than the susceptible strain. Antioxidant enzymes can reduce the damage of reactive oxygen species (ROS) caused by high temperature. Therefore, the antioxidant enzyme genes of *T. truncatus* were screened, and their biological information was analyzed. The expression patterns of antioxidant enzyme genes in two strains of *T. truncatus* under high temperature stress were determined by RT-qPCR method, and their functions were verified by RNA interference (RNAi) technology. The experimental results provide a theoretical basis for understanding the occurrence of agricultural mites under the background of climate warming and applying gene regulation technology to control *T. truncatus* in the future.

**Abstract:**

*Tetranychus truncatus* (Acari: Tetranychidae) has caused serious economic losses on some crops (soybean, corn, and cotton) in China, and has developed resistance to most acaricides. Our laboratory study found that *T. truncatus* was resistant to pyridaben and also adapted to high temperature (34–40 °C). High temperature stress may cause arthropods to produce a large amount of reactive oxygen species (ROS), causing oxidative damage. Antioxidant enzymes, as the main antioxidants, can reduce the damage caused by excessive ROS in arthropods. In order to study the adaptation mechanism of the pyridaben-resistant strain of *T. truncatus* to high temperature and the role of antioxidant enzyme genes under high temperature stress, four antioxidant enzyme genes, *TtSOD*, *TtPOD3*, *TtPOD4*, and *TtGSTs2*, were screened according to the transcriptome sequencing data of pyridaben-susceptible and -resistant strains in *T. truncatus*. Firstly, the phylogeny and structure analyses of these four genes were carried out. Then, real-time quantitative PCR (RT-qPCR) technology was used to analyze the gene expression patterns of antioxidant enzymes in two strains of *T. truncatus* at three different high temperature ranges (34 °C, 38 °C, and 42 °C). The results showed that the expression levels of four antioxidant enzyme genes of two strains of *T. truncatus* were induced by high temperature stress, and the expression levels of antioxidant enzyme genes were significantly different in each development state. The gene expression of antioxidant enzyme genes in resistant strains at the adult stage was significantly higher than that in susceptible strains. After the *TtSOD* and *TtPOD4* genes of adult mites of the resistant strain were silenced by RNA interference (RNAi) technology, the mortality rate of mites with *TtPOD4* gene silencing reached 41.11% after 96 h at 34 °C, which was significantly higher than that of the control and *TtSOD* gene silencing. It has been confirmed that the *TtPOD4* gene plays a key role in the adaptation of pyridaben-resistant strain of *T. truncatus* to high temperature. It lays a theoretical foundation for revealing the thermal adaptation mechanism of *T. truncatus*.

## 1. Introduction

The change in global climate, such as rising temperatures, rising carbon dioxide levels, changing ultraviolet radiation levels, and unpredictable extreme weather events, has had a tangible impact on terrestrial organisms and their interactions. Ectothermic organisms such as insects and other arthropods are particularly vulnerable to global warming; arthropod pests are remarkably capable of rapidly adapting to novel forms of environmental stress [1,2,3].

When arthropods are stressed by adverse or extreme environmental factors, they utilize corresponding adaptive mechanisms in physiology and biochemistry, including regulating stress proteins, producing oxidative stress, or changing other physiological characteristics [4]. For example, *Propylaea japonica* excessive reactive oxygen species (ROS) are generated to inhibit or prevent the oxidative damage caused by an unfavorable environment. Active oxygen in insects mainly include hydroxyl radical (OH-), hydrogen peroxide (H_2_O_2_), and superoxide anion radical (O_2_^−^) [5]. When insects were subjected to high temperature stress, they would produce excessive ROS, which would upset the balance of the original antioxidant process in the body. Excessive ROS will destroy the fluidity of cell membrane, cause cell apoptosis, and lead to lipid peroxidation [6]. Many studies have confirmed that antioxidant enzymes in insects were the most important part of the reactive oxygen species scavenging system, which could alleviate the oxidative damage caused by ROS [7,8,9]. Superoxide dismutase (SOD) was the first line of defense for insects to resist oxidation. It reduces O_2_^−^ to H_2_O_2_, and then decomposes H_2_O_2_ into harmless H_2_O and O_2_ by peroxidase (POD) and catalase (CAT), so as to maintain the balance of active oxygen content in insects [10]. The glutathione -S- transferase (GST) could eliminate the toxic products of lipid peroxidation in cells [11]. For example, Khurshid et al. [12] found that the activities of POD and CAT in *Myzus persicae* were significantly affected after short-term high temperature stress, and the activities of POD and CAT increased significantly with the increase in stress time to prevent oxidative damage caused by the accumulation of reactive oxygen species. GST gene was up-regulated after the adult *Diaphorina citri* was exposed for 15 min at 40 °C [13].

*Tetranychus truncatus* Ehara (Acari: Tetranychidae) is a harmful mite that infests different agricultural fields and greenhouse crops. The mite sucks the juice of plant leaves with a stab-sucking mouthpart, which seriously affects the photosynthesis of plants. It can lead to the reticulation, yellowing, and curling of leaves, thus reducing yield and quality [14]. So far, its distribution is limited to Southeast Asia and East Asia, such as China, Guam, Hainan Island, Indonesia, Japan, Korea, Mariana Island, Philippines, Taiwan, Thailand, and Vietnam [15]. Among spider mites, it is the most economically important species and became the dominant pest in China in 2009. It has a complex diet; many crops have suffered from it, including soybean, corn, and other food crops and vegetables; it even harms jujube, apple, and other fruit trees [16,17]. It is particularly difficult to control mites with conventional acaricides, because of their high fecundity, short life cycle, small size, and strong adaptability to develop resistance quickly, especially in warmer conditions [18]. The resistance to more than 96 acaricides has been reported in *T. urticae* [19]. Pyridaben belongs to mitochondrion electron conduction inhibitor miticide (METI), which targets the NADH coenzyme Q reductase site of complex I and inhibits the mitochondrial electron transfer chain [20]. An H110R mutation was found in the subunit PSST in a drug-resistant strain of *T. urticae*, so it was resistant to pyridaben, tebufenpyrad, and other pesticides [21,22,23].

Studies have found that the high temperature promotes the resistance of arthropods to pesticides [24]. Ge et al. [25] found that when exposed to a high temperature of 40 °C, the mortality rate of *Nilaparvata lugens* treated with triazophos (tzp) decreased significantly (from 94% to 50%), and the long-term extension of LT_50_ was 17.2 h. There is a positive correlation between the resistance of *Laodelphax striatellus* to buprofezin and its heat resistance [26]. The study in our laboratory found that there were significant differences in fertility, longevity, and enzyme activity between pyridaben-susceptible and -resistant strains of *T. truncatus* under high temperature stress, while the pyridaben-resistant strain showed the advantage of adaptability to high temperature [27,28]. We inferred that exposure to pesticide stressors can also induce pests to develop heat tolerance, but the molecular mechanism involved is still unclear. In addition, there is no research on the expression pattern and function of antioxidant enzyme gene in *T. truncatus* under high temperature stress.

Therefore, we sequenced the transcriptome of two strains of *T. truncatus* after high temperature stress at the previous stage [29]. Then, based on the transcriptome data of *T. truncatus* and gene annotation information, we selected four antioxidant enzyme genes (*TtSOD*, *TtPOD3*, *TtPOD4*, and *TtGSTs2*) with significantly up-regulated expression, and analyzed the expression patterns of the four antioxidant enzyme genes in two strains of *T. truncatus* at different developmental stages (egg, larvae, nymph, and adult) under different high temperature stress ranges (34 °C, 38 °C, and 42 °C) by real-time quantitative PCR (RT-qPCR). The function of the highly expressed gene in adult mites was verified by the RNAi method, which laid a theoretical foundation for revealing the molecular thermal adaptation mechanism of its resistant strain. This also provides a theoretical basis for the control of *T. truncatus* by gene regulation technology in the future.

## 2. Materials and Methods

### 2.1. Mite Source

The pyridaben-susceptible strain of *T. truncatus* (Tt-S): It has been kept on the potted cowpea plants in the insect ecological rearing room of the College of Plant Protection of Gansu Agricultural University (L16:D8, 70 ± 5% RH, and 25 ± 1 °C) for about 5 years, and it was not exposed to any acaricides during the rearing period.

The pyridaben-resistant strain of *T. truncatus* (Tt-R): The resistant strain was screened in the laboratory, and the resistance index of pyridaben was 59 times.

### 2.2. High Temperature Stress Treatment

The eggs (600), larvae (500), nymphs (350), and adult females (150) of these two strains of *T. truncatus* were treated at 34 °C, 38 °C, and 42 °C for 1 h, respectively. The spider mites at different developmental stages treated at 25 °C was used as control. After heat treatment, they were collected in an enzyme-free centrifuge tube, and then the samples were quickly frozen in liquid nitrogen and stored in the refrigerator at −80 °C until they were used for RNA extraction. Four biological replicates were set for each treatment.

### 2.3. Sequence and Phylogenetic Analyses of Antioxidant Enzyme Protein

Based on the transcriptome database of *T. truncatus* under high temperature stress (NCBI, Sequence Read Profile (SRA) database, accession numbers from SRR21659835 to SRR21659850), we screened four antioxidant enzyme genes (*TtSOD*, *TtPOD3*, *TtPOD4*, and *TtGSTs2*) with high expression by functional annotation, and the genes were used for subsequent experiments. The amino acid sequences of the screened antioxidant enzyme genes were compared with similar sequences by using the DNAMAN software (v.9.0; LynnonBio-Soft, Quebec, QC, Canada), and then logged into the NCBI gene database, and lastly, the Blast online comparison tool (https://blast.ncbi.nlm.nih.gov/Blast.cgi) (accessed on 12 September 2023) was used to query and screen homologous sequences. SMART software (http://smart.embl-heidelberg.de/) (accessed on 12 September t 2023) was used to predict the signal peptide, catalytic domain, and binding domain of each protein sequence. The pIs and molecular weight of each antioxidant enzyme gene were calculated by using ExPASy Proteomics Server (http://web.expasy.org/protparam/) (accessed on 12 September 2023). Subcellular localization was predicted by Cell-PLoc (http://www.csbio.sjtu.edu.cn) (accessed on 12 September 2023). Then, the phylogenetic tree was constructed by using the neighbor-joining (NJ) method in MEGA 7.0 software. Bootstrap was set at 1000 times. The protein sequence information of antioxidant enzymes of different mites is listed in Table S1.

### 2.4. RNA Extraction and cDNA Synthesis

The samples were taken from the refrigerator at −80 °C for extraction. According to the manufacturer’s instructions, total RNA was extracted using Trizol reagent (Invitrogen, Carlsbad, CA, USA). The concentration of 1 µL RNA was measured by NanoPhotometer-N50 spectrophotometer (GE Healthcare, Wiesbaden, Germany) after RNA was extracted. The OD_260_/_280_ absorption ratio was between 1.80 and 2.20, which indicated that all samples passed the quality assessment. Then, the first-strand cDNAs of samples were synthesized by reverse transcription RNA using the PrimeScript RT Regent Kit with gDNA Eraser (Takara, Dalian, China) and stored in the refrigerator at −20 °C.

### 2.5. Real-Time Quantitative PCR

The expression levels of genes were analyzed using a RT-qPCR approach. Primer 6.0 software was used to design primers for each candidate gene, and β-actin [30] and *RPS18* [31,32] were selected as reference genes. The synthesis of the primer (Table S2) was completed by Guangzhou Qingke Biotechnology Co., Ltd. (Guangzhou, China). The reaction system was carried out by the SYBR Green I chimeric fluorescence method through the fluorescence quantitative PCR instrument (ABI Q5, Applied Bio systems company, Waltham, MA, USA) via the following steps: (1) 95 °C for 2 min, followed by (2) 40 cycles of 95 °C for 15 s, 60 °C for 30 s, and 95 °C for 15 s, and (3) 60 °C for 1 min and 95 °C for 15 s.

The quantitative expression value of the target genes was calculated by using the mathematical model, which simplifies to2^−∆∆CT^ as follows: −∆∆CT = [(CT target − CT reference)_treatment_ − (CT target − CT reference)_control_]. Four biological replications were carried out in each experiment.

### 2.6. RNA Interference

Based on RT-qPCR results, we synthesize dsRNA of of *TtSOD* and *TtPOD4* genes. Using cDNAs cloned previously as templates, the templates of *TtSOD* and *TtPOD4* were amplified by PCR with forward and reverse primers containing the T7 prime sequence TAATACGACTCACTATAGGGG at the 5′ ends (Table S3). The synthesis of dsRNA was performed using Transcript Aid T7 High Yield Transcription Kit (Thermo Scientific, Waltham, MA, USA). The concentration of purified dsRNA was determined by using the NanoPhotometer-N50 spectrophotometer (Implen,, Munich, Germany), and the concentration was about 1000 ng/μL [33].

According to the research method by Yang et al. [34], the specific operation of feeding the gene of *T. truncatus* was as follows: bean leaves were cut into squares of 1.5 × 1.5 cm in size, dried in an oven at 60 °C for 3 min, and treated with 50 μL dsRNA solution at a concentration of 1000 ng/μL for 1–2 h; then, the dsRNA solution was fully absorbed and the leaves were placed in a Petri dish (9 cm in diameter). Fifty female adult mites that were starved for 24 h at room temperature were selected, and the leaves were treated with water as the blank control and GFP as the negative control. After feeding for 48 h, the adult female mites were treated at 34 °C for 1 h; the surviving mites were taken for real-time quantitative PCR and the mean and standard error (mean ± SE) of four replicates were calculated. Mortality was counted every 12 h.

### 2.7. Statistical Analysis

The relative expression of the two strains at different temperatures was analyzed by two-way Analysis of Variance (ANOVA) [35] to test the effects of temperatures, strains, and temperatures × strains with biostatistics software SPSS 23.0 (IBM Corporation, Armonk, NY, USA), and the LSD method was used for post hoc tests (α = 0.05). The relative expression of the antioxidant enzyme gene after silencing was compared using Student’s *t*-test, and the mortality of *T. truncatus* after silencing at 34 °C was compared using the LSD test. Graphs were plotted using OriginPro 2018 (OriginLab Corporation, Northampton, MA, USA).

## 3. Results

### 3.1. Bioinformatics Analysis of Antioxidant Enzyme Genes of T. truncatus

#### 3.1.1. Identification of Antioxidant Enzyme Genes of *T. truncatus*

**Figure 1:**
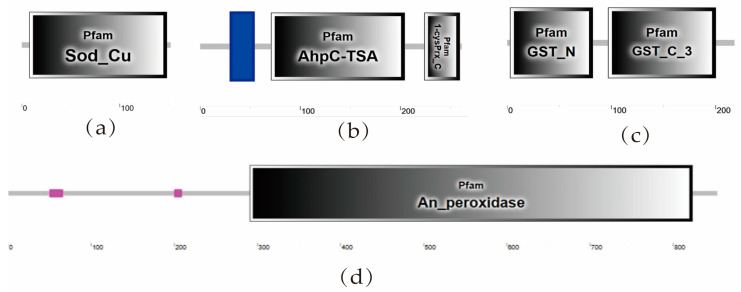
The functional domain of antioxidant enzymes in *Tetranychus truncatus.* (**a**) *TtSOD* gene; (**b**) *POD3* gene; (**c**) *TtGSTs2* gene; (**d**) *POD4* gene. Pink indicates low-complexity region, black indicates catalytic region, and the horizontal line indicates connecting region.

The results of identification of four antioxidant enzyme genes and domain analysis of proteins of T. truncatus are shown in Table 1 and Figure 1.

TtSOD, TtPOD3, TtGSTs2, and TtPOD4 had an open reading frame of 459 bp, 786 bp, 657 bp, and 2562 bp, encoding 152, 261,218, and 853 amino acids, respectively. Predicted molecular weights were 15559.40, 29208.33, 24737.32, and 96085.96 kDa, and their theoretical isoelectric points were 6.02, 7.61, 5.30, and 8.19, respectively (Table 1).

The deduced amino acids contained 140 amino acid Pfam domains (8–148) in the SOD gene. TtPOD3 had a transmembrane structure (31–53) and a Pfam domain (71–204, 224–259) containing 168 amino acids. The deduced amino acids contained 182 amino acid Pfam domains (3–82, 97–200) in the GSTs gene. The TtPOD4 gene included 532 amino acid Pfam domains (291–823) (Figure 1).

#### 3.1.2. Domain Analysis and Sequence Alignment of Antioxidant Enzyme Protein

**Figure 2:**
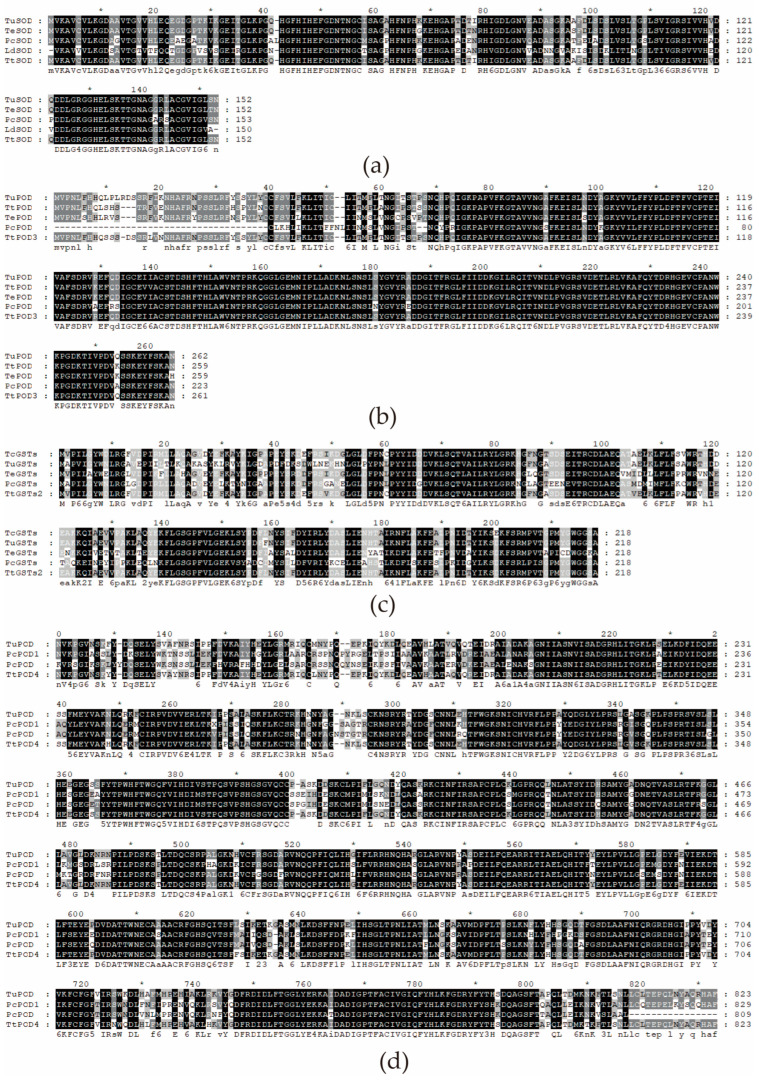
Comparative analysis of amino acid sequences of antioxidant enzyme catalytic domain of *Tetranychus truncatus.* (**a**) *TtSOD* gene; (**b**) *POD3* gene; (**c**) *TtGSTs2* gene; (**d**) *POD4* gene. Tu: *Tetranychus urticae*; Te: *Tetranychus evansi*; Pc: *Panonychus citri*; Tt: *Tetranychus truncatus*. The black part shows the exact same amino acid sequence in all species, and the gray part shows the similar part in most species.

Through multiple sequence alignment of antioxidant enzyme genes in different species, amino acid residues in some positions were highly conserved (Figure 2).

#### 3.1.3. Phylogenetic Analysis of Antioxidant Enzyme

The similarity and identity analysis of four antioxidant genes with other species indicated that the sequence identities of TtPOD4 and TtGSTs2 from *T. truncatus* were 67.43–82.57% and 65.52–96.95%, respectively. *TtPOD3* was 86.22–92.17%, and *TtSOD* was 91.45–97.33%. Among them, *TtSOD*, *TtPOD3*, and *TtPOD4* had the highest sequence identity, reaching more than 92% with *T. urticae* (Table 2). They also had high homology with *T. urticae*.

**Figure 3:**
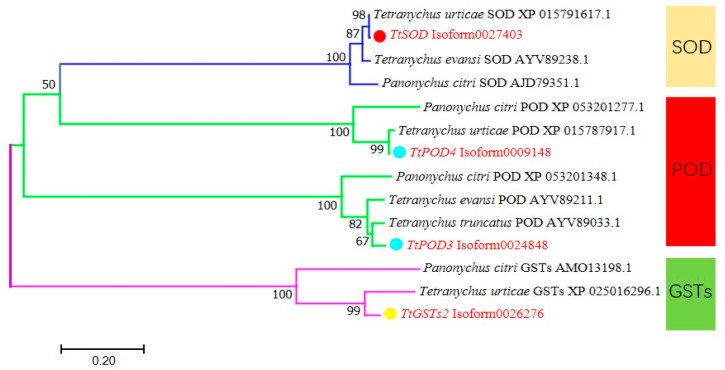
Phylogenetic analysis of antioxidant enzyme catalytic domain of *T. truncatus. TtSOD*, *POD3*, *TtGSTs2*, and *POD4*: four genes in red font.

A constructed phylogenetic tree is shown in Figure 3, where the *TtSOD* and *TtGSTs2* genes of *T. truncates*, and the SOD and GST genes of other mites are clustered on a big branch, respectively. In particular, it has high homology and similarity with *T. urticae*, and it is possible that the two species originated from the same ancestor. *TtPOD3* gene and *TtPOD4* gene are divided into two branches, meaning that *TtPOD3* and *TtPOD4* may have unique functions in different mite species.

### 3.2. Expression Patterns of Antioxidant Enzyme Genes in T. truncatus at Different Temperatures

**Figure 4:**
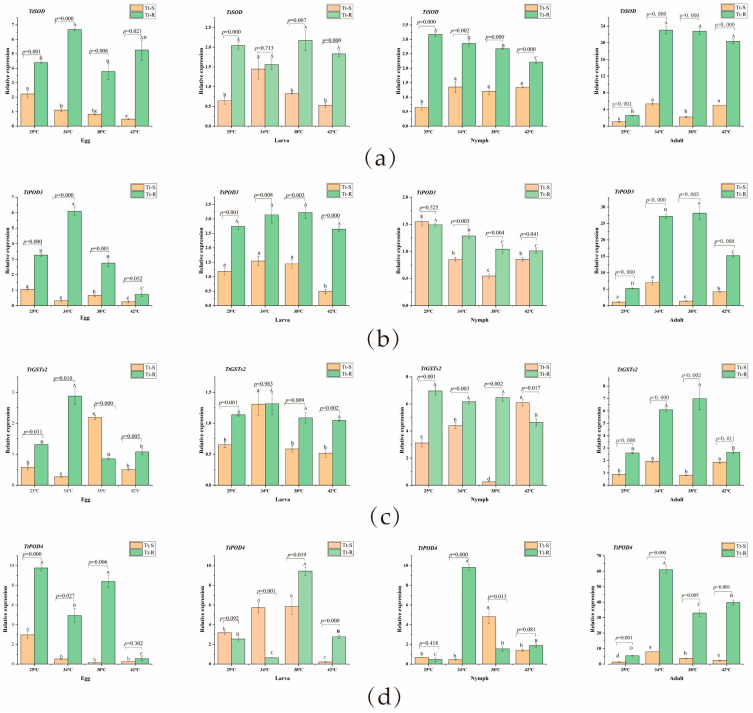
Expression pattern of antioxidant enzyme genes in two strains of *T. truncatus* under high temperature stress. **Tt-S:** susceptible strain of *T. truncatus*; **Tt-R:** pyridaben-resistant strain of *T. truncatus*. (**a**) *TtSOD* gene; (**b**) *POD3* gene; (**c**) *TtGSTs2* gene; (**d**) *POD4* gene. Each value represents the mean ± SE of four repetitions. Different lowercase letters indicate that the gene relative expression of sensitive strains at different temperatures is significantly different (LSD test: *p* < 0.05), and different capital letters indicate that the gene relative expression of resistant strains at different temperatures is significantly different (LSD test: *p* < 0.05). *p* value indicates that there is significant difference in gene relative expression between sensitive and resistant strains under the same thermal stress condition (*p* < 0.05).

As can be seen from Figure 4, four antioxidant enzyme genes were all expressed under high-temperature induction, but the relative expression pattern of every stage of development in *T. truncatus* after heat stress was significantly different between the two strains. Two-way ANOVA analysis of two strains of *T. truncatus* and different high temperatures showed that the four antioxidant enzyme genes were significantly affected by strains and temperature at the adult stage, and there was also a significant interaction between strains and temperature (*F_TtSOD_* = 785.938, *p* < 0.001; *F _TtPOD3_* = 544.284, *p* < 0.001; *F_TtPOD4_* = 501.834, *p* < 0.001; *F_TtGSTs2_*= 237.192, *p* < 0.001). At other immature stages (egg, larva, and nymph), there is also an interaction between temperature and strain (*p* < 0.001).

At each development stage, the expression levels of four antioxidant enzyme genes of the two strains were different at high temperatures. When *T. truncatus* was subjected to different high temperature stresses at the egg stage, the expression levels of *TtSOD*, *TtPOD3*, and *TtGSTs2* genes in resistant strains were significantly up-regulated at 34 °C compared with at room temperature. Their relative expression levels were 1.52 times, 1.87 times, and 2.19 times higher than those in normal temperature, respectively. However, with the increase in temperature, their expression levels decreased significantly. The relative expression levels of *TtSOD*, *TtPOD3*, and *TtPOD4* of susceptible strain decreased significantly during the egg stage.

At the larva stage, the *TtSOD*, *TtPOD3*, and *TtGSTs2* genes of the resistant strain did not change significantly. The *TtSOD*, *TtPOD4*, and *TtGSTs2* genes of susceptible strain increased at 34 °C, and then decreased at 42 °C. The *TtPOD4* gene of the resistant strain changed greatly, which was significantly down-regulated at 34 °C, but significantly up-regulated at 38 °C, reaching 4.41 times as much as that of the normal temperature.

The expression of *TtSOD* gene in resistant strain was down-regulated at nymph stage, while that in susceptible strain was up-regulated at high temperature, but there was no significant difference among different high temperatures. The relative expression of *TtPOD3* gene at the nymph stage of the two strains decreased significantly.

At the adult stage, the relative expression levels of *TtSOD* and *TtPOD4* genes of the two strains increased significantly under different high temperature stresses, and *TtPOD4* gene reached the highest level at 34 °C, which was 6.14 times and 11.34 times higher than that of the normal temperature control group, respectively. The expression levels of *TtPOD3* and *TtGSTs2* genes in resistant strain also increased significantly, but decreased significantly at 42 °C.

### 3.3. Effect of dsRNA Treatment on Mortality of T. truncatus under High Temperature Stress

**Figure 5:**
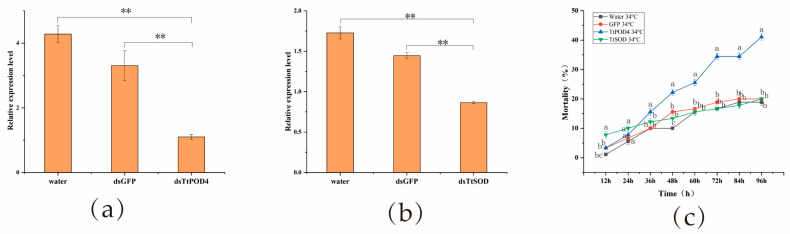
Functional analysis of *TtPOD4* and *TtSOD* of *T. truncatus* by RNA interference followed by high temperature treatment. (**a**,**b**) Relative expression of *POD4* and *TtSOD* after dsRNA-mediated knockdown of *TtPOD4* and *TtSOD* expression. (**c**) Susceptibility of *T. truncatus* to high temperature after knocking down. ** indicates highly significant differences after different RNAi treatments (Student’s *t*-test, *p* < 0.01). Lowercase letters above each line indicate significant differences amongst water, *dsGFP*, *TtPOD4*, and *TtSOD* (LSD test *p* < 0.05).

After the resistant strain of *T. truncatus* was fed the dsRNA of the antioxidant enzymes *TtPOD4* and *TtSOD* at a concentration of 1000 ng/μL for 48 h, the relative expression of the *TtPOD4* and *TtSOD* genes were significantly lower than those of water and dsGFP (Figure 5a,b), and the silencing efficiency rates were 74.29% and 49.95%, respectively. The results showed that dsRNA treatment could effectively inhibit the expression of *TtPOD4* and *TtSOD* genes.

The female adults of *T. truncatus* were treated at 34 °C for 1 h after feeding on the target genes *dsPOD4* and *dsSOD*, and the cumulative mortality of *T. truncatus* at 12 h, 24 h, 36 h, 48 h, 60 h, 72 h, 84 h, and 96 h was observed and counted. There was no significant difference in the mortality of T. truncatus within 24 h after the two genes were disturbed (Figure 5c). With the extension of time, the cumulative mortality rate of *T. truncatus* increased. When *TtPOD4* gene was interfered for 36 h, the mortality of *T. truncatus* was significantly higher than that of *dsSOD* and control, and when *TtPOD4* gene was interfered for 96 h, the mortality reached 41.11%. It was confirmed that the *TtPOD4* gene was involved in the adaptation of the pyridaben-resistant strain of *T. truncatus* to high temperature by RNAi technology.

## 4. Discussion

In the process of evolution, organisms have formed a series of protective mechanisms to adapt to environmental temperature changes. The stress response of antioxidant enzymes is an important strategy for organisms to cope with temperature stress [36]. Antioxidant enzymes play an important role in the process of biological growth and development to mainly maintain the balance of active oxygen metabolism and normal physiological metabolism in organisms [37]. However, different antioxidant enzyme genes were expressed in different expression modes at different stages of organism development. For example, Qin et al. [38] studied the expression pattern of the catalase (CAT) gene of *Frankliniella Occidentalis* at different developmental stages and different temperatures using RT-qPCR technology. The results showed that high temperature stress could induce the expression of CAT, and the expression of CAT in the second-instar nymph of thrips was significantly higher than that in other instars. In this study, we found that high temperature induced the expression of the four antioxidant enzyme genes of *T. truncatus*, and the relative expression level of each developmental state of the two strains was different after heat stress. It was indicated that they were involved in the growth, reproduction, and high-temperature tolerance of mites, but the main functions performed in every stage of developmentof *T. truncatus* may be different. We noticed that there was no significant difference in the expression of *TtPOD3* gene under high temperature stress compared with that under normal temperature, especially at the larva stage. *TtSOD* and *TtGSTs2* genes also showed a similar expression pattern at the larva stage of resistant strain, but the expression level of susceptible strain was high at 34 °C. Also, the relative expression for *TtPOD4* in the susceptible strain is higher than in the resistant strain at 34 °C and 38 °C during both larval and nymph states. We inferred that *TtSOD*, *TtPOD3*, *TtGSTs2*, and *TtPOD4* genes were not involved in the process of resisting high temperature stress at the larva stage, but were more involved in maintaining the normal development of larva. However, eggs may be more sensitive to temperature stress because they cannot move, thus triggering a rapid reaction of antioxidant enzyme genes. In our study, the expression levels of *TtSOD* and *TtPOD3* genes suddenly increased at 34 °C, even reaching the peak at the egg stage, and then gradually decreased with the increase in temperature. Yang et al. [39] found the GST activity of *Panonychus citri* to reach its peak after being exposed for 2 h at 35 °C, and then decreased, which was consistent with the results of our study. This may be because the initial thermal stress led to lipid peroxidation, and the ROS produced could not be effectively removed, thus leading to oxidative stress. In order to protect mites from producing excessive ROS, antioxidant enzyme activity was improved by lipid peroxidation products. With the increase in stress temperature, membrane lipid peroxidation was eliminated, its concentration decreased, and the activity of antioxidant enzyme genes weakened. For example, the red-billed Leiothrix (*Leiothrix lutea*) was subjected to heat stress; its antioxidant defense system was activated to offset the damage caused by high temperature. However, even if the antioxidant level was high, long-term high temperature exposure would cause some degree of oxidative damage, which may take a longer recovery time [40]. Nie et al. [41] also found that heat stress induced oxidative stress, and antioxidant enzymes play an important role in reducing oxidative damage in *T. urticae.* However, antioxidant enzymes were not always enough to offset the production of ROS induced by heat stresses. Female adults of *T. urticae* may have other antioxidant mechanisms, which can protect female adults from the oxidative damage caused by heat stress.

In addition, the relative expression of the *TtSOD* and *TtPOD3* declines during the larval and nymph stages compared with the expression in eggs. This decline is not observed for *TtPOD4*. We inferred that this may be because the function of each antioxidant enzyme is different. In the phylogenetic tree, it was found that the *TtPOD3* gene and *TtPOD4* gene are divided into two branches, which also confirmed this, but their specific functions need to be explored and verified by subsequent experiments.

The relative expression levels of the four antioxidant enzyme genes, *TtSOD*, *TtPOD3*, *TtGSTs2*, and *TtPOD4*, at the adult stage were significantly higher than those at other stages. The conclusions of many studies is consistent with ours. For example, Lu et al. [42] found that the relative expression of PPO, POD, As A-POD, CAT, and Cu/Zn SOD antioxidant enzyme genes in adult *Mononychellus mcgregori* was significantly increased after extreme high temperature stress. The mRNAs of five antioxidant enzyme genes of *Neoseiulus barkeri* were significantly expressed in adults [43]. It was speculated that antioxidant enzyme genes were not only involved in regulating the growth and development at the immature stage, but also in the process of high-temperature resistance or adversity stress at adult stage.

After the *TtPOD4* and *TtSOD* of *T. truncatus* were knocked out, the silencing rate of *TtPOD4* was 74.29%, while that of *TtSOD* was only 49.95%. Therefore, it is speculated that the difference of silencing efficiency of different target genes may be one of the reasons why the mortality of *T.truncatus* with *TtPOD4* gene silencing was significantly higher than that of *TtSOD* gene. In this study, only two antioxidant enzyme genes highly expressed at the adult stage were selected for functional verification. Whether *TtPOD3* or *TtGST* genes are involved in high-temperature tolerance at the egg stage or nymph stage is a speculation that needs further study to confirm or overturn.

In addition, studies show that the transcription factor Nrf2 (nuclear factor (erythroid-derived 2)-like 2) plays a key role in the regulation of antioxidant enzymes in organisms [44,45]. Gao et al. [46] also found that Nrf2 inhibitors could significantly inhibit the gene expression of superoxide dismutase (*TcSOD*), catalase (*TcCAT*), peroxidase (*TcPOD*), and polyphenol oxidase (*TcPPO*) in Tetranychus cinnabarinus. From the transcription level, it was confirmed that the expression of transcription factor *TcNrf2* affects the expression of downstream antioxidant enzyme. Could the transcription factor Nrf2 regulate the *TtPOD4* resistant strain of *T. truncatus*? Further research is needed to explore this issue.

We found that the relative expression of the antioxidant gene in the pyridaben-resistant strain was higher than that of susceptible strains at the adult stage under different high temperature stresses. Ge et al. [25] found that triazophos induced the heat tolerance of *Nilaparvata lugens* by increasing gene expression, that is, arthropods exposed to pesticide stressors could have cross-tolerance to another stressor (high temperature). Wang et al. [47] found that high temperature can induce the expression of CYP 450 gene in *Liriomyza trifolii* and increase its tolerance to pesticides. It can be seen that high temperature and pesticides can trigger the cross-resistance of arthropods to exogenous stressors. But some studies have shown subtle differences. For example, at 35 °C or higher, the tolerance of *Bemisia tabaci* biotype (BTQ) to thiamethoxam decreased, while the moderate high temperature of 31 °C increased the tolerance to thiamethoxam [24]. Even some studies have found that pesticide-resistant strains of insects have no adaptability to high temperature. The wing vein damage rate of the resistant strain of *Plutella xylostella* was higher than that of susceptible strain under high temperature stress; the survival rate was also lower than that of susceptible strain [48]. The adaptability of the chlorpyrifos-resistant strain of *Nilaparvata lugens* to higher temperature is lower than that of susceptible strain [49]. This indicates that some organisms have made an adaptive trade-off between heat and pesticide. These different results show the complexity of molecular and biochemical reactions when individuals face multiple pressures. Therefore, only by fully understanding the impact of external pressure sources on pests can we control them more effectively. In addition, our experiment is only aimed at the *T. truncatus* resistant strains to a single insecticide. Do other resistant strains also show adaptation to high temperature? What factors are related to the adaptability of pesticide strains to high temperature or the fitness trade-off? All of these need further study.

## 5. Conclusions

In summary, we identified and described four antioxidant enzyme genes from *T. truncatus*. These genes contained the Pfam domain and were highly conserved. High temperature stress can induce the expression of antioxidant enzyme genes of *T. truncatus*. The expression mode of antioxidant enzyme genes in different developmental states were different after heat stress. The expression of the *TtPOD4* gene was higher at the adult stage of two strains of *T. truncatus*. The expression levels of *TtSOD* and *TtPOD3* genes in resistant strains were higher at the egg stage and adult stage, while those in sensitive strains were higher only at adult stage. The expression levels of *TtGSTs2* gene at nymph stage and adult stage of both strains were higher. The relative expression levels of resistant strains were higher than those of susceptible strains at the adult stage at different temperatures. The *TtPOD4* gene was involved in the adaptation of the pyridaben-resistant strain of *T. truncatus* to high temperature.

## Figures and Tables

**Table 1:** Information about antioxidant enzyme genes from *T. truncatus*.

Genes	Gene ID	Ref Seq mRNA	ORF	aa	Molecular Weight (kDa)	Theoretical pI	Protein Subcellular Localization Prediction
*TtSOD*	Isoform0027403	XP_015791617.1	459	152	15,559.40	6.02	Nucleus
*TtPOD3*	Isoform0024848	AYV89033.1	786	261	29,208.33	7.61	Cytoplasm
*TtGSTs2*	Isoform0026276	XP_025016296.1	657	218	24,737.32	5.30	Cytoplasm
*TtPOD4*	Isoform0009148	XP_015787917.1	2562	853	96,085.96	8.19	Cytoplasm

Note: ORF (open reading frame), aa (amino acid).

**Table 2:** The analysis of the similarity and identity of four antioxidant genes with other species.

Gene Names	Species	Accession Number	Similarity (%)	Identity (%)
*TtSOD*	*Tetranychus urticae*	XP_015791617.1	100%	97.33%
	*Tetranychus evansi*	AYV89238.1	100%	96.71%
	*Panonychus citri*	AJD79351.1	100%	91.45%
*TtPOD3*	*Tetranychus urticae*	AYV89033.1	100%	92.72%
	*Tetranychus evansi*	AYV89211.1	100%	90.04%
	*Panonychus citri*	XP_053201348.1	85%	86.22%
*TtPOD4*	*Tetranychus urticae*	XP_015787917.1	100%	96.95%
	*Panonychus citri*	XP_053201277.1	99%	65.52%
*TtGSTs2*	*Tetranychus urticae*	XP_025016296.1	100%	82.57%
	*Panonychus citri*	AMO13198.1	100%	67.43%

## Data Availability

All data supporting this research are included in this article.

## Supplementary Materials

The following supporting information can be downloaded at: https://www.mdpi.com/article/10.3390/insects15060381/s1, Table S1: The protein sequence information of antioxidant enzymes of different mites; Table S2: Primer sequences of RT-qPCR for *T. truncatus*; Table S3: Primer sequences of RNAi for *T. truncatus*.
