# Supplementary material for: Differential Antioxidant Enzyme Gene Expression and Functional Analysis of Pyridaben-Susceptible and -Resistant Strains of Tetranychus truncatus (Acari: Tetranychidae) under High Temperature Stress
Source: Insects. 2024 May 23;15(6):381. doi: 10.3390/insects15060381 (PMC11204104; doi:10.3390/insects15060381)
Supplement: Supplementary file 1 [file insects-15-00381-s001.zip › insects-2982592-supplementary.pdf]

Table S1 The protein sequence information of antioxidant enzymes of different mites

Species	Protein name	Accession number	Phylogenetic group
Tetranychus urticae	Superoxide dismutase	XP_015791617.1	Acariformes
Tetranychus evansi	Superoxide dismutase	AYV89238.1	Arachnida
Panonychus citri	Superoxide dismutase	AJD79351.1	Acariformes
Tetranychus truncatus	Peroxiredoxin	AYV89033.1	Acariformes
Tetranychus evansi	Peroxiredoxin	AYV89211.1	Arachnida
Panonychus citri	Peroxiredoxin	XP_053201348.1	Acariformes
Tetranychus urticae	Peroxiredoxin	XP_015787917.1	Acariformes
Panonychus citri	Peroxiredoxin	XP_053201277.1	Acariformes
Tetranychus urticae	Glutathione-S-transferase	XP_025016296.1	Acariformes
Panonychus citri	Glutathione-S-transferase	AMO13198.1	Acariformes

Table S2 Primer sequences of RT-qPCR for *T. truncatus*

Gene name	Primer name	Primer sequence（5′-3′）
RPS18	*RPS18-F*	ACGTGCTGGTGAACTTACCGAAGA
	RPS18-R	TGCCTATTCAAGAACCAAAGTGGG
Actin	*Actin-F*	GCCATCCTTCGTTTGGATTTGGCT
	Actin-R	TCTCGGACAATTTCTCGCTCAGCA
TtSOD	*TtSOD-F*	TGGTAAAAGCTGTCTGTGTT
	TtSOD-R	TATGTGGATTGAAATGGGCT
TtPOD3	*TtPOD3-F*	TACCTTCGTCTGTCCCACTG
	TtPOD3-R	GTCCCCGGAAAGTGATACCA
TtPOD4	*TtPOD4-F*	GTGAACAATCCTGCGGCTAA
	TtPOD4-R	TTGATGTTGAAGGCGATTCTGT
TtGSTs2	*TtGSTs2-F*	TCCAGCTCCTGAGTATTCCAA
	TtGSTs2-R	AATCCATGTTTCCTTCCCAAGT

Table S3 Primer sequences of RNAi for T. truncatus

Gene name	Primer name	Primer sequence（5′-3′）
TtSOD	*dsTtSOD-F*	TAATACGACTCACTATAGGGGTCTGTGTTCTTAAAGGAG
	dsTtSOD-R	TAATACGACTCACTATAGGGCTGATAATGGACCAGTAAG
TtPOD4	*dsTtPOD4-F*	TAATACGACTCACTATAGGGCTGATAGTGAGAAGAGAC
	dsTtPOD4-R	TAATACGACTCACTATAGGGGGAGGAATCTTAGTCAAG
GFP	*dsGFP-F*	TAATACGACTCACTATAGGGCAGTTCTTGTTGAATTAGATG
	dsGFP-R	TAATACGACTCACTATAGGGTTTGGTTTGTCTCCCATGATG
